# An Environmental *Escherichia coli* Strain Is Naturally Competent to Acquire Exogenous DNA

**DOI:** 10.3389/fmicb.2020.574301

**Published:** 2020-09-03

**Authors:** Francesco Riva, Valentina Riva, Ester M. Eckert, Noemi Colinas, Andrea Di Cesare, Sara Borin, Francesca Mapelli, Elena Crotti

**Affiliations:** ^1^Department of Food, Environmental and Nutritional Sciences (DeFENS), University of Milan, Milan, Italy; ^2^Molecular Ecology Group, National Research Council - Water Research Institute (CNR-IRSA), Verbania, Italy; ^3^Institut Cavanilles de Biodiversitat i Biologia Evolutiva, Universitat de València, Valencia, Spain

**Keywords:** antibiotic resistance, horizontal gene transfer, treated wastewater, rhizosphere, root colonization, *E. coli* genomes, One Health

## Abstract

The diffusion of antibiotic resistance determinants in different environments, e.g., soil and water, has become a public concern for global health and food safety and many efforts are currently devoted to clarify this complex ecological and evolutionary issue. Horizontal gene transfer (HGT) has an important role in the spread of antibiotic resistance genes (ARGs). However, among the different HGT mechanisms, the capacity of environmental bacteria to acquire naked exogenous DNA by natural competence is still poorly investigated. This study aimed to characterize the ability of the environmental *Escherichia coli* strain ED1, isolated from the crustacean *Daphnia* sp., to acquire exogenous DNA by natural competence. Transformation experiments were carried out varying different parameters, i.e., cell growth phase, amount of exogenous DNA and exposition to artificial lake water (ALW) and treated wastewater to mimic environmental-like conditions that may be encountered in the agri-food system. Results were compared with those showed by the laboratory *E. coli* strain DH5α. Our experimental data, supported by genomic sequencing, showed that, when exposed to pure water, ED1 strain was able to acquire exogenous DNA with frequencies (10^–8^–10^–9^) statistically higher than the ones observed for DH5α strain (10^–10^). Interestingly, higher values were retrieved for ED1 than DH5α strains exposed to ALW (10^–7^ vs. 10^–9^, respectively) or treated wastewater (10^–8^ vs. 10^–10^, respectively). We tested, therefore, ED1 strain ability to colonize the rhizosphere of lettuce, a model plant representative of raw-consumed vegetables of high economic importance in the ready-to-eat food industry. Results showed that ED1 strain was able to efficiently colonize lettuce rhizosphere, revealing a stable colonization for 14 days-long period. In conclusion, ED1 strain ability to acquire exogenous DNA in environmental-like conditions by natural competence, combined with its ability to efficiently and stably colonize plant rhizosphere, poses the attention to food and human safety showing a possible route of diffusion of antibiotic resistance in the agri-food system, sustaining the “One Health” warnings related to the antibiotic spread.

## Introduction

Antibiotic Resistance (AR) is a public concern for global health. About 700,000 people die every year from antibiotic resistant bacteria-infections and 10 million annual deaths caused by antibiotic resistant pathogens are estimated by 2050 (Lim et al., 2019). In the last century, antibiotics have been widely used in medicine, plant production and livestock industries, imposing a strong selective pressure on the environmental microbial communities (Van Hoek et al., 2011). The exposition of bacteria to a sub-lethal concentration of antibiotics has led to the generation and diffusion of antibiotic resistant bacteria (ARB), through mutations and horizontal gene transfer (HGT) of antibiotic resistance genes (ARGs) (Smalla et al., 2018). This can be particularly enhanced in specific hot spots of natural and engineered ecosystems, such as mycosphere, residuesphere, rhizosphere and wastewater treatment plants (WWTPs) (Eckert et al., 2018; Riva et al., 2020). The spread of ARGs in different environments linked to anthropogenic activities has been largely demonstrated: for example, long-term applications of sewage sludge and chicken manure can improve the abundance and the diversity of ARGs and ARB in soil (Chen et al., 2016), while WWTPs can be considered as one of the main ARGs’ contaminated aquatic systems for both ARB and free DNA (Czekalski et al., 2014; Amos et al., 2015). Despite several studies have described the presence and spread of ARGs and ARB in the environment, some gaps of knowledge about the selection, evolution, persistence and HGT of ARGs remain to be unveiled (Larsson et al., 2018; Smalla et al., 2018).

HGT is crucial for bacterial adaptation to new environments and, consequently, for bacterial evolution. DNA transfer is generally accomplished by three “classical” mechanisms, namely transduction, conjugation and transformation (Sun, 2018). While in transduction and conjugation specific apparatuses are required to transfer DNA from donor to recipient cells, i.e., phage virions and conjugative pili, respectively, in transformation the acquisition of DNA is usually transient and linked to the capability of the bacterial cells to express competence at a specific physiological phase. Concerning the environmental ARG diffusion through HGT mechanisms, researchers have highlighted that many aspects have yet to be clarified, e.g., the contribution of the different mechanisms to ARG spread or the drivers of gene transfer (Smalla et al., 2018). For instance, since conjugation-based experiments are more feasible in laboratory and field conditions than those based on the other HGT mechanisms, this might have underestimated the importance of transformation or transduction (Smalla et al., 2018).

Natural competence for transformation is a specific physiological state in which bacteria are able to acquire genetic material from their surroundings. The acquired DNA can be then integrated into the bacterial genome or be maintained as a plasmid in the cell (Blokesch, 2016). There are more than 80 prokaryotic species described to be naturally transformable and different species and strains can show peculiar traits: for instance, *Vibrio cholerae* has been described to acquire DNA in presence of chitin (Meibom et al., 2005), while *Acinetobacter baylyi* is constitutively competent for transformation with frequency rates depending on the bacterial growth phase (Blokesch, 2016; Domingues et al., 2019). For a long time, *Escherichia coli* has not been considered a naturally transformable bacterium. *E. coli* is routinely forced to acquire exogenous DNA by artificial laboratory treatments, i.e., following the exposure to (i) solutions with high concentrations of divalent metal ions followed by heat shock, (ii) polyethylene glycol solutions, or (iii) electrical shock pulses (Hasegawa et al., 2018). Nonetheless, in some specific conditions, not related to the artificial transformation, *E. coli* has been demonstrated capable to acquire exogenous DNA, e.g., in contact with environmental waters (Baur et al., 1996; Woegerbauer et al., 2002; Ishimoto et al., 2008), in food extracts (Maeda et al., 2003) or after freeze-thaw processes. Besides the “classical” exogenous DNA uptake machinery of natural transformation, based on conserved proteins for the transport of single-stranded DNA (ssDNA) into the cell cytoplasm, two new routes of DNA acquisition by transformation have been recently identified in this species. In the first way, double-stranded DNA (dsDNA) is internalized into *E. coli* cells on agar plates, while the second DNA uptake mechanism depends on a cell-to-cell contact, not related to conjugation, and occurs in a colony on agar plates (Sun et al., 2006, 2009; Etchuuya et al., 2011; Sun, 2018). While the latter mechanism has been recently reported to be induced by a P1*vir* bacteriophage (Sugiura et al., 2017), the former foresees the participation of several proteins, among which researchers have so far identified *ydcS* and *ydcV* genes, encoding for a putative periplasmic protein and a putative inner membrane protein, respectively (both located on the putative ABC transporter *ydcSTUV* operon for putrescine transport; Sun, 2016) and the general stress response regulator factor RpoS (Zhang et al., 2012; Sun, 2016).

One of the main recognized routes that could allow AR spread in environments related to the agri-food system is the use of reclaimed water for irrigation purposes. Nowadays the water reuse represents a common practice in several countries and is considered a priority also by the European water management policy to combat the water crisis exacerbated by global warming (Riva et al., 2020). Indeed, at least 20 million hectares of croplands worldwide are irrigated with urban treated wastewater (Bouaroudj et al., 2019). WWTPs have been indicated as one of the main contributors of both cell bound and free ARGs for the aquatic systems (Czekalski et al., 2014; Amos et al., 2015; Li et al., 2018; Zhang et al., 2018); the reuse of treated wastewater for irrigation purposes would enter the food production and could contribute to the diffusion of ARGs that finally could potentially be acquired by pathogenic strains. Indeed, it has been found that WWTPs can promote, in the water in which the effluents are released, the stabilization of a resistome derived principally from treated wastewaters (Corno et al., 2019), making the freshwater bodies reservoirs of ARGs (Di Cesare et al., 2015). The ability of *E. coli* to acquire and transfer exogenous DNA (Hasegawa et al., 2018; Sun, 2018), together with its capability to survive and thrive in different habitats (i.e., water, rhizospheric soil or human gut; Van Elsas et al., 2011; Raimondi et al., 2019), where the presence of ARGs has been reported (Du et al., 2020; Osińska et al., 2020) and HGT can be enhanced (i.e., rhizosphere, Chen et al., 2019), could pose a risk for the food safety and public health (Krzeminski et al., 2019). This risk could be high for fresh products such as spinach, sprout, and lettuce, which are generally consumed as raw vegetables (Shen et al., 2019). Indeed, antibiotic resistant bacteria belonging to the pathogenic species *E. coli* and *Salmonella enterica* have been already reported in farm environments and fresh products, including lettuce and ready-to-eat food (Nüesch-Inderbinen et al., 2015; Araújo et al., 2017; Schierstaedt et al., 2019; Perera et al., 2020; Yang et al., 2020).

In the framework of “One Health” approach, this study aimed to (i) characterize the possible acquisition of exogenous DNA by an environmental strain of *E. coli* mimicking the conditions that may be encountered in the agri-food system, and to (ii) study the *E. coli* strain capacity to colonize plant rhizosphere, using soil potted lettuce as model system.

## Materials and Methods

### Strains and Media

*Escherichia coli* strain ED1 was isolated from individuals of *Daphnia* sp. collected from a small rainwater-fed pond in the garden of the CNR-IRSA, Verbania, Italy. Thirty daphnids (in triplicates) were washed in sterile Milli-Q water, crushed and sonicated (3 times, 1 min each cycle with a shaking application by vortexing between cycles) in 1 ml of 2M NaCl. Serial 10-fold dilutions were prepared and filtered on nitrocellulose membrane filters (type GSWP, 25 mm diameter, 0.22 μm pore size, Millipore) which were placed onto agar plates of the selective medium mFC (Biolife) and incubated for 24–48 h at 37°C. Once colonies of presumptive *E. coli* (blue color on mFC agar) appeared on plates, they were purified by streaking three times and then stored in 25% glycerol solutions at −80°C. A small amount of the bacterial biomass was then introduced in 1 ml of 2M NaCl, centrifuged (5000 g, 10 min, 4°C), boiled 15 min, frozen for 2–4 h and finally centrifuged as before. One of the isolate, named ED1, was identified as an *E. coli* strain due to positive amplification of the *uid*A gene (Srinivasan et al., 2011) by PCR as described elsewhere (Sabatino et al., 2015).

### Preparation of Transforming Exogenous DNA

Transformations were carried out by using pCR^TM^II-TOPO^®^ (Invitrogen) plasmid carrying ampicillin and kanamycin resistance genes. The plasmid was extracted from the strain *E. coli* Mach1^TM^ T1 Phage-Resistant pCR^TM^II-TOPO^®^ using the QIAPrep^®^ Spin Miniprep Kit (Qiagen, Milan, Italy) following the manufacturer instructions. The plasmid was quantified by measuring the optical density at 260 nm wavelength in a spectrophotometer (BIO RAD SmartSpec^TM^ 3000).

### Natural Transformation Protocol

Precultures of ED1 and DH5α strains were firstly grown in 25 ml of LB liquid medium overnight at 37°C with shaking. Then, 1 ml of cultures were diluted in 100 ml of LB and incubated at 37°C until the cells reached early exponential or stationary growth phases, i.e., at optical densities at 600 nm (OD_600 nm_) of 0.4–0.5 or 2, respectively. Forty milliliter of cells were then centrifuged twice with Milli-Q water for 10 min at 2700 g and finally resuspended in 500 μl of the same washing buffer. All centrifugation steps were performed at room temperature (RT) between 20 and 23°C. Four aliquots of 100 μl of cells were prepared, and the proper quantities of plasmidic DNA were added and gently mixed, without pipetting (the mixture is hereafter named as transformation mixture). The remaining 100 μl-cells aliquot was used as negative control (no DNA was added). Samples were incubated at RT for 1 h: three aliquots were then plated on LB plates added with ampicillin (100 μg/ml), while the fourth aliquot was serially diluted (from the undiluted sample to -8) and used to evaluate (in triplicate) the total cell count on LB agar plates without the antibiotic selection. Aliquot of negative control was plated as well on LB plates added with ampicillin (100 μg/ml). All the plates were kept at 37°C overnight. Experiments were performed with three biological replicates. Putative colonies of transformants, retrieved by ampicillin selection, were then streaked on LB plates added with kanamycin (100 μg/ml). Both ED1 and DH5α strains are sensitive to 100 μg/ml ampicillin, 100 μg/ml kanamycin and 50 μg/ml rifampicin. To further confirm the plasmid acquisition, kanamycin-resistant colonies were also subjected to PCR amplification. Transformation frequencies were calculated as the ratio between the number of transformants and the total number of culturable cells (about 10^9^ cell/ml in case of cells harvested at the exponential phase and 10^10^ cell/ml in case of cells harvested at the stationary phase). Bacterial transformation was performed using 0.25, 0.5, 1, and 2 μg of plasmidic DNA.

Transformation protocols were then carried out using cells collected at the early exponential phase and exposing them to 2 μg of plasmidic DNA in two different types of water as washing and incubation buffers: besides Milli-Q water (pH 6.23) we used (i) artificial lake water (ALW, pH 7.69) prepared modifying the protocol of Zotina et al. (2003) in regard to the inorganic medium components (Supplementary Table 1), and (ii) water collected from the effluent of a WWTP located in Verbania (pH 6.84; water sampled on December 10th, 2019; Supplementary Table 1), serving 51,000 population equivalent and equipped with chlorination as disinfection process (Di Cesare et al., 2016). In order to reduce the presence of environmental bacteria, water samples were filtered through nitrocellulose membrane filters with 0.22 μm pore size (Millipore).

DNase sensitivity was tested by adding DNase I to the transformation mixture at different times, e.g., immediately after the transformation mixture preparation and after 1, 3, 4, 6, and 18 h from the preparation of the transformation mixture. Then, the transformation mixtures containing DNase I were incubated 1 h at RT before plating on LB agar plates added with ampicillin (100 μg/ml) (Sun et al., 2006).

To verify the acquisition of pCR^TM^II-TOPO^®^ plasmid, DNA was extracted from putative transformants by boiling lysis (Ferjani et al., 2015) and used as template to amplify a plasmid sequence fragment of about 250 bp with primer M13f (-20) (5′-GTA AAA CGA CGG CCA G-3′) and M13r (5′-CAG GAA ACA GCT ATG AC-3′) according to manufacturer’s (Invitrogen) instruction. Thermal protocol was set up as follows: 94°C for 5 min, followed by 34 cycles at 94°C for 1 min, 55°C for 1 min and 72°C for 1.5 min and the last step at 72°C for 10 min.

### Generation of Rifampicin Resistant Mutants of *E. coli* Strains ED1 and DH5α

Rifampicin mutants of ED1 and DH5α strains were obtained by plating stationary-phase cultures on LB plates added with 50 μg/ml of rifampicin. Plates were then incubated at 37°C overnight. Upon appearance, rifampicin resistant (RIF-R) colonies were selected and initially re-streaked on LB added with 50 μg/ml rifampicin and, finally, on LB added with 100 μg/ml rifampicin.

### Root Colonization by *E. coli* Strains

RIF-R ED1 and RIF-R DH5α strains were used for the bacterization of *Lactuca sativa* (var. Canasta) seedlings to verify their ability to colonize plant rhizosphere. Lettuce seeds were sterilized with 0.7% sodium hypochlorite for 5 min followed by 5 rinsing steps in sterile distilled water (Bonaldi et al., 2015) and grown in pots filled with non-sterile soil under greenhouse conditions. Three days after sowing, lettuce seedlings (*n* = 3 for each strain) were inoculated with 5 ml of bacterial suspensions obtained by growing the RIF-R ED1 and RIF-R DH5α strains in LB medium supplemented with rifampicin (100 μg/ml) for 24 h at 37°C, centrifuging twice the bacterial cultures at 4000 rpm for 10 min and re-suspending the pelleted cells in physiological solution (NaCl 0.9%) to obtain a final bacterial concentration of 10^8^ cell/g of soil. Six lettuce seedlings were irrigated with 5 ml of distilled water and considered as negative control. One week after bacterization, lettuce seedlings were harvested and the rhizosphere soil was separated from the root by vortexing for 5 min the root system in physiological solution. To evaluate the number of colony-forming units (cfu) per gram of soil, rhizosphere samples (*n* = 3 for each strain; *n* = 6 for negative control) were serially diluted in physiological solution, plated in triplicate on LB medium supplemented with rifampicin (100 μg/ml) and cfu were counted after 24 h of incubation at 30°C. In order to confirm the identity of the isolates, after the visual check of colony morphology on the Petri dishes, 10 bacterial colonies isolated from the rhizosphere of each bacterized lettuce seedlings were picked. The DNA was extracted through boiling cell lysis and the 16–23S rRNA Intergenic Transcribed Spacer (ITS) region was amplified by ITS-PCR fingerprinting (Mapelli et al., 2013), comparing the ITS profiles of the bacteria re-isolated from the rhizosphere at the end of the experiment with those of RIF-R ED1 and RIF-R DH5α strains used for lettuce bacterization.

The colonization experiment was repeated to investigate the stability of ED1 and DH5α strains in the lettuce rhizosphere over time (14 days). For this experiment, lettuce seeds were sterilized as reported above and grown in soil previously sterilized through tindalization process. One week after sowing, lettuce seedlings were inoculated with 5 ml of bacterial suspensions (10^8^ cell/g of soil) prepared as described above. The presence of RIF-R ED1 and RIF-R DH5α strains in lettuce rhizosphere was verified 1 week (t1) and 2 weeks after bacterization (t2). As previously described, rhizosphere soil samples (*n* = 3 for each strain and each experimental time) were serially diluted and plated in triplicate on LB medium supplemented with rifampicin (100 μg/ml). Assessment of cfu/g of soil and strain identity were performed as described above.

### DNA Extraction, Genome Sequencing, and Analysis

Genomic DNA from *E. coli* strain ED1 was extracted from an overnight culture in LB liquid medium using the UltraClean Microbial DNA extraction kit (Qiagen), according to the manufacturer’s protocol. DNA quantity was assessed using fluorometry (Qubit, Invitrogen) according to the manufacturer’s protocol. Sequencing was performed on an Illumina NovaSeq platform using paired-end sequencing of 150 bp fragments at IGA Technologies (Udine, Italy). The genome was assembled as described by Cabello-Yeves et al. (2018): briefly, *Trimmomatic* was used for read trimming and filtering and *SPAdes* for the genome assembly, while a preliminary gene annotation was done using NCBI (Johnson M. et al., 2008). This Whole Genome Shotgun project has been deposited at DDBJ/ENA/GenBank under the accession JAAWVB000000000. The version described in this paper is version JAAWVB010000000.

Genome assemblies of *E. coli* strains ED1 and K12 NEB DH5α (Accession Number CP017100; Anton and Raleigh, 2016) were submitted to the RAST Service^1^ and compared taking advantage of the RAST function-based comparison tool. Plasmid presence in ED1 genome was investigated through the platform PlasmidFinder (Carattoli et al., 2014)^2^. VirulenceFinder 2.0 platform (Joensen et al., 2014)^3^ was used to identify virulence genes in the genomes of *E. coli* strains ED1, K12 NEB DH5α, O157:H7 Sakai (Accession Number BA000007, Makino et al., 1999) and O157:H7 EDL933 (Accession Number AE005174, Perna et al., 2001). Genomic islands, insertion sequences (IS) and phage genome sequences were searched in ED1 and K12 NEB DH5α genomes by IslandViewer4 (Bertelli et al., 2017), ISfinder (Siguier et al., 2006) and PHASTER (Arndt et al., 2016). Details on RAST and NCBI annotation can be found in Supplementary Table 2.

### Statistical Analyses

Statistical analyses were conducted with R 3.1.2 (R Core Team, 2013) through RStudio (RStudio Team, 2015) and with Calc Statistical Function of Microsoft^®^ Office Excel. Linear model was applied to assess the relation between transformation frequency and quantities of DNA added during transformation protocols. Student’s *t*-test was employed to verify differences between ED1 and DH5α strains concerning transformation frequencies (considering growth phase and types of water) and root colonization efficiency.

## Results

### Influence of Different Growth Phases on Transformation

**FIGURE 1:**
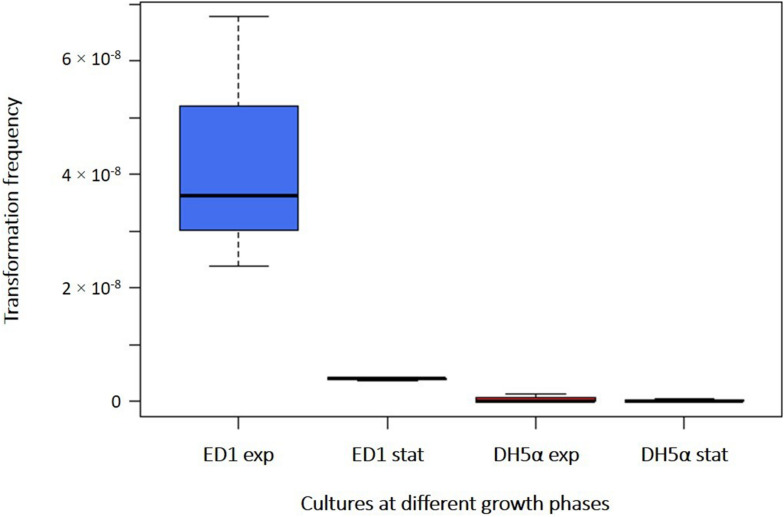
Transformation frequencies of *E. coli* strains ED1 and DH5α with cells collected at early exponential (“exp”) and stationary (“stat”) phases. Transformations were performed in Milli-Q water with 2 μg of plasmidic DNA.

The capability to acquire exogenous DNA by the environmental *E. coli* strain ED1, compared with the laboratory *E. coli* strain DH5α, was initially tested in pure water on resting cells harvested at different phases of the growth curve: Milli-Q water was used as washing and incubation buffer (to avoid the presence of interfering cations) and a large amount of transforming DNA plasmid (2 μg) was added to minimize any possible interference on transformation frequencies linked to a limiting quantity of DNA. First, we used cells harvested from early exponential phase cultures (OD_600 nm_ between 0.4 and 0.5) (Supplementary Figure 1), observing a transformation frequency of 4.26 × 10^–8^ (± 2.26 × 10^–8^) and 4.44 × 10^–10^ (± 7.70 × 10^–10^) for ED1 and DH5α strains, respectively (Supplementary Table 3). ED1 cells in early exponential growth phase demonstrated a significantly higher transformation frequency in comparison with DH5α cells (Student’s *t*-test, *p* = 0.032, Figure 1). When cells were harvested at the stationary phase (OD_600 nm_ between 2.1 and 2.2), a transformation frequency of 3.95 × 10^–9^ (± 3.91 × 10^–10^) was obtained with ED1 strain, resulting however statistically higher than the value recovered for DH5α strain (1.93 × 10^–10^ ± 1.56 × 10^–10^; Student’s *t*-test, *p* = 0.0001, Figure 1 and Supplementary Table 3). While transformation frequencies of DH5α strain were not significantly different between both growth phases (Student’s *t*-test, *p* = 0.609), statistical analysis indicated that ED1 natural competence is significantly higher in the early exponential phase than in the stationary one (Figure 1; Student’s *t*-test, *p* = 0.0415). All the following transformation assays were therefore run with cells at the early exponential phase.

In order to confirm the occurrence of natural transformation (which is a DNase-sensitive mechanism differently from the DNase-resistant mechanisms i.e., conjugation and transduction), we checked the sensitivity of ED1 uptake of DNA to the addition of DNase I. Since no transformation events were retrieved, unveiling thus the DNase sensitivity of the mechanism, we confirmed ED1 cells’ ability to uptake DNA by natural competence (Hasegawa et al., 2018).

### Influence of Exogenous DNA Quantity on Transformation Frequency

**FIGURE 2:**
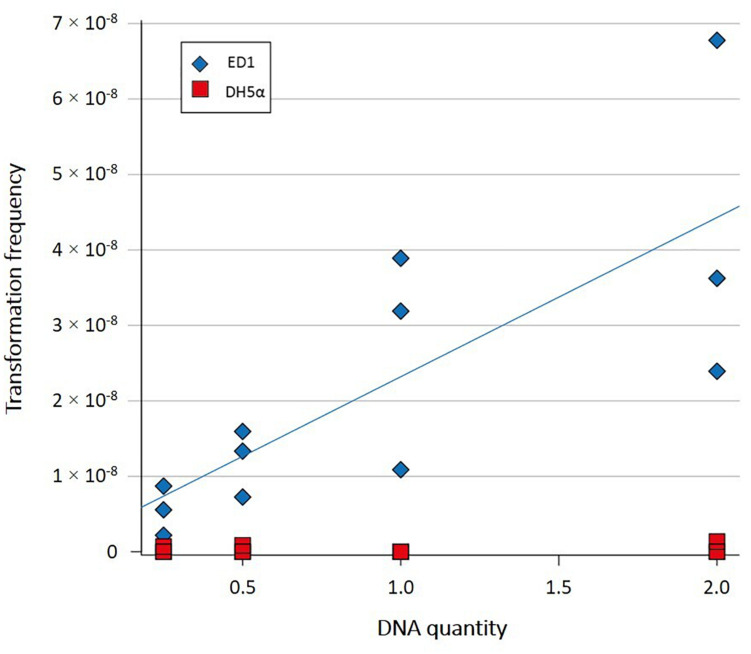
Transformation frequencies of *E. coli* strains ED1 and DH5α in Milli-Q water with increasing quantities of plamidic DNA. Transformation frequency of ED1 strain was significantly related to the amount of plasmid added (linear model: *t* = 3.9, *p* = 0.003).

Transformation frequencies of ED1 and DH5α strains were analyzed in Milli-Q water with increasing quantities of plasmid pCR^®^ II-TOPO^®^ as exogenous DNA, by adding 0.25, 0.5, 1, and 2 μg of plasmidic DNA to the cells harvested at the early exponential phase. As shown in Supplementary Table 4, transformation frequency for DH5α strain was estimated to be ≤ 4.44 × 10^–10^, while increasing transformation frequencies were reported for ED1 strain, ranging from 5.48 × 10^–9^ to 4.26 × 10^–8^ when increasing quantities of plasmid from 0.25 to 2 μg, respectively, were added. Statistical analysis revealed a statistical difference for ED1 strain exposed to 2 or 0.25 μg of plasmidic DNA (Student’s *t*-test, *p* = 0.0480 between 2 and 0.25 μg). As shown in Figure 2, transformation frequency of ED1 strain was significantly related to the amount of plasmid added (linear model: *t* = 3.9, *p* = 0.003), whereas this was not the case for DH5α strain (linear model: *t* = 0.55, *p* = 0.6) (Baur et al., 1996).

### Bacterial Transformation in Different Types of Waters

**FIGURE 3:**
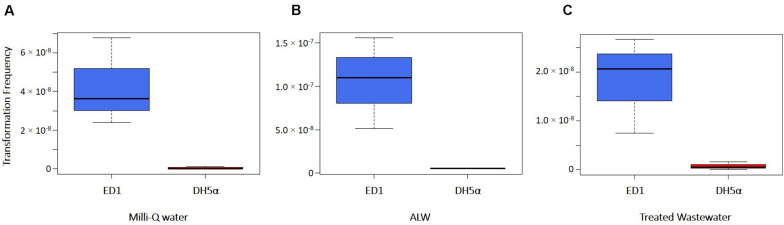
Transformation frequencies of *E. coli* strains ED1 and DH5α using different types of water: **(A)** Milli-Q water, **(B)** ALW, and **(C)** treated wastewater (WW).

Transformation of ED1 and DH5α strains was assessed in natural and artificial water solutions considered as representative of environmental habitats, i.e., the artificial lake water (ALW) and the water collected from the effluent of Verbania WWTP. Milli-Q water was used as control and the transformations were carried out with a not limiting quantity of transforming DNA (2 μg). Statistical analysis showed that the transformation frequencies of ED1 strain were significantly higher than the ones observed for DH5α strain considering all the types of water used (Student’s test; *p* = 0.0295, 0.0226, and 0.0364 with ALW, Milli-Q water and treated wastewater, respectively, Figure 3). Transformation frequencies ≤ 5.19 × 10^–9^ were obtained for DH5α strain in the different types of water (Supplementary Table 5). Moreover, transformation frequencies of ED1 strain were significantly higher in ALW than in the other types of water (Figure 3; Student’s *t*-test *p*-values: between Milli-Q water and ALW, *p* = 0.029; between ALW and treated wastewater, *p* = 0.047): specifically, we obtained for this strain transformation frequencies values of 1.06 × 10^–7^ (± 5.26 × 10^–8^) in ALW and 1.83 × 10^–8^ (± 9.80 × 10^–9^) in the effluent water released into the environment from Verbania WWTP, whereas for the control in pure water a value of 4.26 × 10^–8^ (± 2.26 × 10^–8^) was retrieved (Supplementary Table 5).

### Plant Colonization by *E. coli* Strains

**FIGURE 4:**
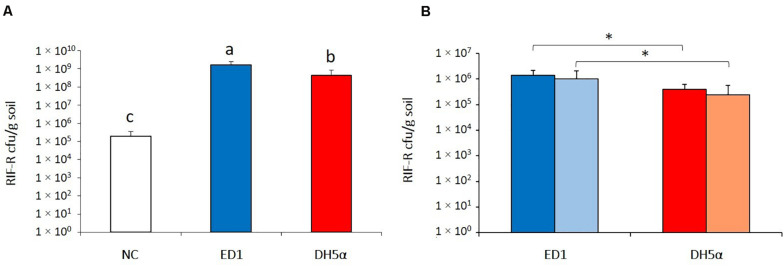
Evaluation of *E. coli* strains ED1 and DH5α rhizocompetence. **(A)** Bacterial abundance (cfu/g soil) of rifampicin-resistant bacteria in the lettuce rhizosphere after a 1-week colonization experiment performed with non-sterile soil. **(B)** Assessment of the stability of the rifampicin-resistant bacteria abundance in the rhizosphere of lettuce seedlings grown in sterile soil. Dark- and light-colored columns indicate cfu/g of soil 1 and 2 weeks after the bacterization, respectively. NC, non-bacterized lettuce seedlings; ED1/DH5α, lettuce seedlings bacterized with ED1 and DH5α strains, respectively. Different letters or asterisks indicate statistically significantly differences according to Student’s *t*-test (*p* < 0.01).

The ability of ED1 and DH5α strains added to the soil to colonize plants’ rhizosphere was verified using the correspondent RIF-R strains and lettuce seedlings as model system. The experiment was firstly conducted in short term conditions in non-sterile soil to check the rhizocompetence of *E. coli* strains in presence of the competing soil dwelling microbial community. Seven days after *E. coli* addition to the 3 days-old plantlets surrounding soil, the rifampicin resistant bacteria re-isolated from the rhizosphere of the lettuce seedlings amounted to 1.59 × 10^9^ (± 8.29 × 10^8^) cfu/g rhizospheric soil for RIF-R ED1 strain and resulted statistically higher in comparison to rifampicin resistant bacteria isolated from the rhizosphere of both non-bacterized lettuce seedlings (1.97 × 10^5^ ± 1.62 × 10^5^cfu/g soil; *p* = 1.61 × 10^–7^) and seedlings bacterized with RIF-R DH5α strain (4.23 × 10^8^ ± 4.45 × 10^8^ cfu/g soil; *p* = 1.14 × 10^–3^), as shown in Figure 4A. Ten randomly picked colonies isolated from each bacterized plant (*n* = 30 per ED1 strain bacterization; *n* = 30 per DH5α strain bacterization) were subjected to ITS-PCR fingerprinting. The ITS profiles detected for all colonies corresponded to those of the *E. coli* strains used for plants bacterization, as shown in Supplementary Figure 2A and Supplementary Figure 2B for ED1 and DH5α, respectively. Although both the tested *E. coli* strains were able to colonize in 7 days the lettuce rhizosphere under non-sterile soil condition, the environmental *E. coli* strain ED1 showed a higher colonization performance of this microhabitat compared to the laboratory strain DH5α (*p* = 1.92 × 10^–3^).

Similar results were obtained when the experiment was repeated with lettuce plants older (7 days-old) than those used in the first colonization assay in sterile soil and for a longer period, to verify the stability of the strains in the rhizosphere microhabitat, without any competition with the soil residing microbiota. As shown in Figure 4B, 1 week after plant bacterization with ED1 strain, 1.35 × 10^6^ (± 8.39 × 10^5^) cfu/g soil of RIF-R cells were recovered, whereas plants exposed to DH5α strain led to isolate from the lettuce rhizosphere a significant lower RIF-R titer (3.86 × 10^5^ ± 2.38 × 10^5^ cfu/g soil; *p* = 0.0043). The RIF-R isolated colonies in all the assays demonstrated to belong to the inoculated *E. coli* strains by evaluating their ITS-PCR fingerprinting on representative colonies (Supplementary Figure 3). Two weeks after bacterization the presence of both ED1 and DH5α *E. coli* strains remained stable in plants rhizosphere, amounting, respectively to 1.01 × 10^6^ (± 1.03 × 10^6^) cfu/g soil and 2.44 × 10^5^ (± 3.33 × 10^5^) cfu/g soil (*p* = 0.048; Figure 4B).

### Genome Analysis

Taking advantage of RAST function-based comparison tool, genomes of *E. coli* strains ED1 and K12 NEB DH5α (Anton and Raleigh, 2016; Accession Number CP017100), composed of 5,159,712 and 4,583,637 bp, respectively, were compared. *E. coli* strain K12 NEB DH5α has been chosen as reference strain for genomic analysis since it is a fhuA2 derivative of *E. coli* K12 DH5α, the genome sequence of which is not currently available.

Both genomes shared a high percentage of metabolic functions: indeed, the presence of all the main basic metabolic functions, such as, for instance, carbohydrate metabolism or respiration, was assessed. Differences in the genomes’ size are primary reflected in the fact that ED1 strain owns a larger number of genes (included in the below reported metabolic pathways) than K12 NEB DH5α strain. We detected the presence of the “propanediol metabolic pathway” and the “methylcytrate cycle” in ED1 genome, which were absent in K12 NEB DH5α genome (Table 1). Furthermore, in ED1 genome we retrieved genes encoding proteins related to the osmoregulatory choline-glycine betaine system e.g., the high-affinity choline uptake protein BetT, a choline dehydrogenase and a betaine aldehyde dehydrogenase (Table 1). Only in the genome of ED1 we found several genes classified by RAST as involved in the bacterial adhesion and secretory systems, i.e., CFA/I pili, the secretion system type I and the type III secretion injectosome (Table 1).

**TABLE 1:** Main subsystems revealed in the genome of *E. coli* strain ED1.

**Role**	CDS ID in ED1 genome*	**ED1**
IncF conjugative transfer genes	HBA78_21285-HBA78_21370, HBA78_21380-HBA_21390, HBA78_21405-HBA78_21425, HBA78_21435-HBA78_21455,	+
Propapendiol metabolism pathway	HBA78_16485-HBA78_16525, HBA78_16535-HBA_7816560, HBA78_16570-HBA78_16580	+
Type I secretion system LapB, C, E	HBA78_15000, HBA78_15005, HBA78_ 15015	+
CFA/I fimbriae encoding system	HBA78_08810, HBA78_08825	+
Type III secretion system	HBA78_04615, HBA78_04630, HBA78_04645, HBA78_04655-HBA78_ 04665, HBA78_04680, HBA78_04690, HBA78_04695, HBA78_20315, HBA78_ 20335	+
Choline and Betaine Uptake and Betaine Biosynthesis	HBA78_08680, HBA78_08695	+
Hydroxyaromatic non-oxidative decarboxylase protein	HBA78_20830, HBA78_20835	+

“+” indicates presence of functions; *Details on RAST and NCBI annotation can be found in Supplementary Table 2.

Considering genes related to the acquisition of exogenous DNA, the automatic annotation revealed in both genomes the presence of several genes homologous to those required for the DNA uptake in species that are known to be naturally competent: *pilQ/HofQ* (HBA78_15695 and NEB5A_17330; HBA78 code refers to ED1 strain, while NEB5A one refers to K12 NEB DH5α strain), encoding for a transmembrane channel allowing dsDNA to cross the outer membrane; *pilA* (HBA78_09875; NEB5A_00545), *pilB* (HBA78_09880 and NEB5A_00540), *pilC* (HBA78_09885; NEB5A_00535), related to the construction of the pseudopilus; *dprA* (HBA78_16130; NEB5A_16795), also called *smf*, responsible of the DNA processing and *ycaI/ComEC* (HBA78_03445; NEB5A_04210) related to the uptake of exogenous DNA (Chen and Dubnau, 2004; Cameron and Redfield, 2006; Sun, 2018). We detected in both genomes the presence of genes involved in one of the two *E. coli*-specific mechanisms of natural transformation, i.e., the general stress response regulator factor RpoS (HBA78_20820; NEB5A_05530) (Zhang et al., 2012; Sun, 2018), as well as the RpoS-regulated genes *ydcS* and *ydcV* (HBA78_24185 and HBA78_24200) in ED1; NEB5A_07355 and NEB5A_07370 in K12 NEB DH5α) (Sun, 2016). Additional analysis was performed submitting ED1 genome to the PlasmidFinder platform (Carattoli et al., 2014; Yang et al., 2015; Table 1): we found the presence of i) a IncFII plasmid replicon sequence (with an identity of 96.55% against the one of the reference sequence AY458016) and ii) a IncX1 plasmid replicon sequence (with an identity of 95.23% against the one of the reference sequence JN935898). The replicon sequences were located on two separate contigs of 79,647 and 25,889 bp, respectively, and allowed us to speculate the presence of two plasmids in ED1 chromosome.

In order to identify virulence factors, we further analyzed the genomes of strains ED1 and K12 NEB DH5α through the platform VirulenceFinder 2.0 (Joensen et al., 2014). We found a higher number of virulence factors in ED1 than in DH5α genome (Table 2). Both genomes showed the presence of the glutamate decarboxylase (GAD) system which contributes to acid resistance in the human gut (Vanaja et al., 2009). Conversely, we detected only in ED1 genome the presence of genes encoding the adhesin *air*, an enteroaggregative immunoglobulin repeat protein involved in bacterial aggregation and colonization (Sheikh et al., 2006), *astA*, a heat stable enterotoxin-1 (Yatsuyanagi et al., 2003) and *eilA*, a putative activator of the type three secretion system (T3SS), which contributes to the pathogenicity of enteroaggregative *E. coli* (EAEC) strains (Sheikh et al., 2006). Moreover, from the comparison with DH5α genome we found that ED1 genome lost *iss* virulence factor, defined as a serum survival gene (Johnson T.J. et al., 2008). When we included in our analysis the genomes of two pathogenic strains of *E. coli*, i.e., *E. coli* strains O157:H7 Sakai (Accession Number BA000007, Makino et al., 1999) and O157:H7 EDL933 (Accession Number AE005174, Perna et al., 2001), we could observe that a conspicuous higher number of virulence factors was retrieved in the latter than in ED1 or K12 NEB DH5α genomes (Supplementary Table 6). Whereas similar numbers of genomic islands are present in both genomes, the number of IS sequences predicted in ED1 genome is higher than the one retrieved for NEB DH5α genome. Moreover, we found more phage genomic sequences in the former than in the latter strain (Supplementary Table 7).

**TABLE 2:** Virulence genes revealed by the analysis of the genomes of *E. coli* strains ED1 and K12 NEB DH5α using the platform VirulenceFinder 2.0.

		**ED1**	**K12 NEB DH5α**
*air*	Enteroaggregative immunoglobulin repeat protein	+	−
*astA*	Heat stable enterotoxin-1	+	−
*eilA*	hilA-like regulator in enteroaggregative *E. coli*	+	−
*gad*	Glutamate decarboxylase	++	++
*iss*	Increased serum survival	*−*	+

*“+” indicates presence of functions; “−” indicates absence of functions. Number of “ + ” indicates the number of sequences of virulence factors detected in genomes.*

## Discussion

Several studies have revealed the modest capability of *E. coli* strains to acquire exogenous DNA by natural transformation and researchers have recently underlined the existence of a few peculiar DNA uptake mechanisms of natural transformation in this species (Sun et al., 2006; Guo et al., 2015; Hasegawa et al., 2018). *E. coli* laboratory strains, known for their high artificial transformation efficiency, demonstrated to undergo to natural transformation in experiments mimicking natural conditions, e.g., using freshwater or food extracts (Baur et al., 1996; Woegerbauer et al., 2002; Maeda et al., 2003, 2004), whereas a limited number of publications verified natural competence in *E. coli* strains isolated from human and warm-blooded animals (Tsen et al., 2002; Woegerbauer et al., 2002; Matsumoto et al., 2016). Environmental *E. coli* strains, to our knowledge, were never tested for natural competence. In this study, we investigated the ability of the environmental *E. coli* strain ED1, isolated form the crustacean *Daphnia* sp., to acquire exogenous DNA, comparing the results with the ones showed by the laboratory *E. coli* strain DH5α in relation to the cell growth phase, amount of transforming DNA and in environmental-mimicking conditions, i.e., exposed to lake water and WWTP effluents.

We ascertained a higher transformation frequency (10^–8^–10^–9^) for the environmental strain than for the laboratory one (10^–10^), observing a higher number of transformation events when high quantities of plasmidic DNA were used, up to a saturation level (Baur et al., 1996). Values retrieved for ED1 strain underlined the modest capability of transformation in *E. coli* strains, especially if compared with other bacterial strains known to be naturally competent, such as *Acinetobacter baylyi* BD413 (Lorenz et al., 1992) and *Bacillus subtilis* 168 (Hauser and Kanamata, 1994). As reported by Baur et al. (1996), our results showed higher transformation frequencies for ED1 strain with cells grown at early exponential growth phase (0.4–0.6 OD_600 nm_) rather than at the stationary one. Log-phase cells were also used by Woegerbauer et al. (2002) who compared the transformation frequency and efficiency of laboratory and clinical isolates, revealing higher transformation rates for the former. In case of DH5α strain we recovered low values of transformation frequencies: we retrieved only two transformants in all the replicates in which Milli-Q water and 2 μg of DNA were applied. Nevertheless, other studies reported in case of DH5α strain higher numbers of transformants or transformation frequencies than the ones we obtained, likely due to differences of the adopted experimental protocols, which included, among the others, variations of the bacterial growth condition and growth phase (Woegerbauer et al., 2002; Sun et al., 2006).

The protocol we adopted in our experiments was conceived to mimic conditions feasible in the environment. To this aim, strains were subjected to a few manipulation procedures before incubation on selective agar plates and were exposed to different kinds of waters considered as representative of a few habitats (i.e., ALW and treated wastewater). Moreover, temperatures of 20–23°C, closer to environmental values than the ones usually used in laboratory procedures, were maintained during the transformation protocol (not for the incubation), differently from what reported in literature i.e., 37°C (Sun et al., 2006), 10°C or temperature shifts (Baur et al., 1996). Although it was reported that disinfection by-products in the WWTP effluents can enhance the rate of bacterial transformation, promoting the spread of extracellular ARGs (Augsburger et al., 2019; Mantilla-Calderon et al., 2019; Jin et al., 2020; Lu et al., 2020), ED1 strain showed higher transformation efficiency in presence of ALW than treated wastewater. This could be due, on one hand, to a water composition of ALW that was more similar to that of the original habitat of the bacterium; on the other, lower transformation frequencies detected for ED1 strain in presence of treated water than ALW could be related to the peculiar chemical composition of the sampled water (Pereira et al., 2015; Papageorgiou et al., 2016). Thus, we cannot rule out that experiments performed with water collected in different moments could bring the same results. Certain natural and anthropic environments could supply optimal conditions for natural transformation. An example are biofilms in which cell density is very high and cells can be exposed to high concentrations of free DNA (even higher than the ones routinely used in laboratory procedures) derived from the dead neighboring cells (Baur et al., 1996; Hasegawa et al., 2018); this condition can result in ARGs acquisition and spread in the bacterial communities, as characterized in several studies (Petrovich et al., 2018). Moreover, clinically relevant ARGs enter freshwater systems through the outflow of WWTPs (Zhang et al., 2018).

Gram-positive and Gram-negative bacteria that are known to be naturally transformable usually share a similar DNA uptake machinery linked to the Type IV pili and Type II secretion systems (Claverys and Martin, 2003) and both ED1 and K12 NEB DH5α showed the presence of these genes in their genomes. Taking into account the peculiar *E. coli* DNA uptake machineries (Sun, 2018), we found the presence of the genes encoding the transcriptional regulator RpoS that regulates *E. coli* natural transformation (Zhang et al., 2012), as well as the RpoS-regulated genes *ydcV* and *ydcS*, which are involved in the DNA internalization into the inner membrane (Sun, 2016). Although we retrieved in both *E. coli* strain genomes the presence of the above-mentioned genes, we demonstrated that ED1 transformation frequency was higher than DH5α one. Even though we observed an overall genomic function-based similarity between the strains (using the RAST function-based comparison tool), we cannot exclude the existence of some signaling-dependent or regulatory mechanisms that can favor natural transformation in ED1 rather than in DH5α strain. Natural transformation is known to be a very complex mechanism activated differently among species and strains (Lorenz and Wackernagel, 1994; Blokesch, 2016). For instance, in *Haemophilus influenzae* natural competence was demonstrated to be triggered by a lack of phosphotransferase system (PTS) sugars and purine precursors (Mell and Redfield, 2014). Furthermore, since only a DNA-based analysis has been performed in our study, we do not have information about the effective production of the proteins corresponding to the natural transformation-related genes.

Genomic analysis allowed to identify a larger number of genes encoding for metabolic pathways in ED1 genome rather than in the one of K12 NEB DH5α strain, e.g., we found in ED1 genome the propanediol utilization pathway, which allows *E. coli* to grow in anaerobic conditions using rhamnose as carbon source (Liu et al., 2007) and the genes of methilcytrate cycle, which allows microorganisms to use propionate as a carbon/energy sources, being especially useful in the propionate-rich environments such as the gastrointestinal tract (Upton and McKinney, 2007). Furthermore, we found several genes that may help ED1 to thrive in different habitats, i.e., genes encoding for proteins related to the osmotic stress (involved in the synthesis and uptake of compatible solutes; Sim et al., 2014); genes involved in cell to cell aggregation and biofilm production, such as RTX that seems to be responsible for cell-surface adhesions, cells’ aggregation and production of biofilm (Tchagang et al., 2018); CFA/I pili-related genes implicated in the bacterial adhesion through the production of fimbriae; and genes encoding the type III secretion injectosome (Diepold et al., 2011; Zheng et al., 2019; Table 1). Therefore, strain ED1 has different traits that may help it to thrive in the environment and that might be related to a high transformation rate success. Moreover, the higher total amount of the mobile genetic elements found in ED1 than in K12 NEB DH5α could be due to the fact that these elements are commonly found in bacteria exposed to a “horizontal gene pool”, which can be easily found in several environments (Dobrindt et al., 2004). HGT is, indeed, known to contribute to bacterial adaptation to different habitats and, in the long term, to bacterial evolution (Lorenz and Wackernagel, 1994; Thomas and Nielsen, 2005; Vandecraen et al., 2017). This result is also in agreement with the data available on *Vibrio* species, the transformation proficiency of which appears to be more common in environmental strains than in clinical ones (Bernardy et al., 2016).

The environments where *E. coli* is known to survive include soil, water and manure besides several micro-habitats associated to plants, given the ability of some *E. coli* strains to colonize roots, leaf surfaces and endosphere (Van Elsas et al., 2011; Wright et al., 2017; Eissenberger et al., 2020). The capacity of an environmental and naturally transformable *E. coli* strain like ED1 to survive in soil and colonize the plant rhizosphere has relevant implications in the light of the antibiotic cycle and the One Health vision. The plant rhizosphere is indeed a well characterized, substrate-rich, hot spot for bacterial activity and abundance (Zhu et al., 2018), where naturally competent cells can find higher concentrations of free DNA and could, moreover, reach the growth phase in which transformation occurs with high frequency (Sørensen and Jensen, 1998; Mølbak et al., 2003; Ling et al., 2016; Zhu et al., 2018). Relevant concentrations of ARGs can reach the plant rhizosphere, e.g., through soil amended with manure, sewage sludge and treated wastewater (Chen et al., 2017; Riva et al., 2020; Wu et al., 2020). We selected lettuce as a model plant for the root system colonization experiments, as representative of raw-consumed vegetables of high economic importance in the ready-to-eat food industry. Our results showed that ED1 strain colonized efficiently the lettuce rhizosphere both in sterile and non-sterile soils and indicated that the rhizosphere colonization was stable over a period of 14 days. The ability of ED1 strain to acquire exogenous DNA in environmental mimicking conditions and to efficiently colonize the plant rhizosphere might represent a possible route of ARGs spread in the plant microbiome, potentially representing a risk for health through the consumption of raw vegetables (Nüesch-Inderbinen et al., 2015). In this perspective we analyzed the ED1 strain genome for the presence of virulence factors, revealing a higher number of virulence factors in this environmental and naturally competent strain than in the laboratory strain K12 NEB DH5α. Although further analyses are required to unveil any possible relation with human pathogenic *E. coli* strains, these data allow us to hypothesize a low and not relevant virulence for *E. coli* strain ED1 (Supplementary Table 6).

## Conclusion

We demonstrated the ability of an environmental *E. coli* strain to acquire exogenous DNA by natural competence with relatively high frequency in exponential growth phase in environmental-like conditions, together with its capability, when applied to soil, to thrive in lettuce rhizosphere. These results confirm the importance to further investigate the possible spread of antibiotic resistant determinants through HGT in the environment and, particularly, in the rhizosphere of those plant species consumed as raw vegetables, to elucidate the related food and human safety risks. Further studies on environmental *E. coli* strains could allow to strengthen our results and to understand the spread of this phenomenon.

## Data Availability Statement

The datasets presented in this study can be found in online repositories. The names of the repository/repositories and accession number(s) can be found below: https://www.ncbi.nlm.nih.gov/genbank/, JAAWVB000000000.

## Author Contributions

EC, FM, and SB designed the study. FR, VR, EE, NC, and AD carried out the experiments. FR, VR, FM, and EC analyzed the data. FM and SB supported the research. FR and EC wrote the first draft of the manuscript. All authors contributed to the manuscript revision, read and approved the submitted version.

## Conflict of Interest

The authors declare that the research was conducted in the absence of any commercial or financial relationships that could be construed as a potential conflict of interest.

## Footnotes

1 http://rast.nmpdr.org/

2 https://cge.cbs.dtu.dk/services/PlasmidFinder/

3 https://cge.cbs.dtu.dk/services/VirulenceFinder/
