# Supplementary material for: An Environmental Escherichia coli Strain Is Naturally Competent to Acquire Exogenous DNA
Source: Front Microbiol. 2020 Sep 3;11:574301. doi: 10.3389/fmicb.2020.574301 (PMC7494812; doi:10.3389/fmicb.2020.574301)

Supplementary Material

An environmental *Escherichia coli* strain is naturally competent to acquire exogenous DNA

Francesco Riva[1], Valentina Riva[1], Ester M. Eckert[2], Noemi Colinas[2,3], Andrea Di Cesare[2], Sara Borin[1], Francesca Mapelli[1], Elena Crotti[1]

[1]Department of Food, Environmental and Nutritional Sciences (DeFENS), University of Milan, Milan, Italy

[2]Molecular Ecology Group, National Research Council - Water Research Institute (CNR-IRSA), Verbania, Italy

[3]Institut Cavanilles de Biodiversitat I Biologia Evolutiva, Universitat de Valencia, Valencia, Spain

Correspondence:
Dr. Elena Crotti
elena.crotti@unimi.it

SUPPLEMENTARY TABLES

Supplementary Table 1. Chemical composition of a) water collected from the effluent of a WWTP located in Verbania (sampled on December 10th, 2019) and b) ALW (modified from Zotina et al., 2003).

a) Parameter	WWTP effluent
pH	6.84
COD	7 ppm
BOD5	<5 ppm
Total suspended solids	<4 ppm
Total N	6.26 ppm
NH4	0,19 ppm
NO2	<0,02 ppm
NO3	5,27 ppm
Cl^-	38 ppm
$SO4^{2-}$	18,03 ppm
Conductance	292 microsiemens
b) Parameter	ALW
pH	7.69
$NaHCO_3$	168 ppm
NH_4Cl	1.65 ppm
KNO_3	3 ppm
$MgSO_4$	49.3 ppm
K_2SO_4	0.07 ppm
$CaCl_2$	5.88 ppm

Supplementary Table 2. Details on RAST and NCBI annotation for genes of interest included in Table 1 (*see excel file).* Column A indicates the locus_tag of each gene analysed in ED1 genome. Column B and column C indicate RAST and NCBI Prokaryotic Genome Annotation Pipeline annotation, respectively. Columns D and E indicate the results of blasting aminoacidic sequences against the NCBI public database (non-redundant protein sequences) by BLASTp tool and their Accession numbers, respectively.

Supplementary Table 3. Transformation frequencies of *E. coli* strains ED1 and DH5α in Milli-Q water with 2 µg of plasmidic DNA at different growth phases (early exponential and stationary phases). "REP.B" and "REP.T" are biological and technical replicates, respectively. "NTC" indicates number of transformed colonies observed in each technical replicate; "TF" is the average transformation frequency (in brackets standard deviations are indicated).

			Early exponential phase		Stationary phase[a]	
	REP.B	REP.T	NTC	TF	NTC	TF
ED1	1	A	8		10	
		B	12		33	
		C	13		10	
	2	A	14	4.26×10^{-8} ($\pm 2.26 \times 10^{-8}$)	7	3.95×10^{-9} ($\pm 3.91\times 10^{-10}$)
		B	22		20	
		C	25		60	
	3	A	15		13	
		B	17		50	
		C	18		3	
DH5α	1	A	0		0	
		B	0		0	
		C	0		3	
	2	A	0	4.44×10^{-10} ($\pm 7.70 \times 10^{-10}$)	3	1.93×10^{-10} ($\pm 1.56\times 10^{-10}$)
		B	0		7	
		C	0		0	
	3	A	0		0	
		B	1		3	
		C	1		0	

[a]Total cfu/ml at the stationary phase was one order of magnitude higher than the cfu/ml measured at the exponential phase.

Supplementary Table 4. Transformation frequencies of *E. coli* strains ED1 and DH5α in Milli-Q water measured by applying an increasing quantity of plasmid. "REP.B" and "REP.T" are biological and technical replicates, respectively. "NTC" indicates number of transformed colonies observed in each technical replicate; "TF" is the average transformation frequency (in brackets standard deviations are indicated).

			µg DNA*							
			0.25		0.5		1		2	
strain	REP.B	REP.T	NTC	TF	NTC	TF	NTC	TF	NTC	TF
ED1	1	A	5	5.48×10^{-9} ($\pm 3.26 \times 10^{-9}$)	7	1.22×10^{-8} ($\pm 4.46 \times 10^{-9}$)	10	2.72×10^{-8} ($\pm 1.46 \times 10^{-8}$)	8	4.26×10^{-8} ($\pm 2.26 \times 10^{-8}$)
		B	4		5		21		12	
		C	3		10		13		13	
	2	A	1		5		10		14	
		B	3		4		8		22	
		C	1		3		17		25	
	3	A	1		5		6		15	
		B	0		0		4		17	
		C	2		5		5		18	
DH5α	1	A	0	2.22×10^{-10} ($\pm 3.85 \times 10^{-10}$)	0	2.78×10^{-10} ($\pm 4.81 \times 10^{-10}$)	0	0	0	4.44×10^{-10} ($\pm 7.70 \times 10^{-10}$)
		B	0		0		0		0	
		C	0		0		0		0	
	2	A	0		0		0		0	
		B	0		1		0		0	
		C	0		0		0		0	
	3	A	0		0		0		0	
		B	0		0		0		1	
		C	1		0		0		1	

*DNA final concentration in the transformation mixture was 2.1, 4.2, 8.3 and 16.7 µg/ml for a final DNA quantity of 0.25, 0.5, 1 and 2 µg, respectively.

Supplementary Table 5. Transformation frequencies of *E. coli* strains ED1 and DH5α in Milli-Q water, ALW and treated wastewater (WW) following the addition of 2 µg of plasmidic DNA to cells harvested at the early exponential growth phase. "REP.B" and "REP.T" are biological and technical replicates, respectively. "NTC" indicates number of transformed colonies observed in each technical replicate; "TF" is the average transformation frequency (in brackets standard deviations are indicated).

			Milli-Q		ALW		Treated WW	
strain	REP.B	REP.T	NTC	TF	NTC	TF	NTC	TF
ED1	1	A	8		187		2	
		B	12		22		5	
		C	13		39		25	
	2	A	14	4.26×10^{-8} ($\pm 2.26 \times 10^{-8}$)	95	1.06×10^{-7} ($\pm 5.26 \times 10^{-8}$)	0	1.83×10^{-8} ($\pm 9.80 \times 10^{-9}$)
		B	22		54		0	
		C	25		49		9	
	3	A	15		12		7	
		B	17		13		11	
		C	18		12		15	
DH5α	1	A	0		3		1	
		B	0		6		0	
		C	0		2		0	
	2	A	0	4.44×10^{-10} ($\pm 7.70 \times 10^{-10}$)	2	5.14×10^{-9} ($\pm 3.42 \times 10^{-10}$)	2	7.02×10^{-10} ($\pm 8.04 \times 10^{-10}$)
		B	0		1		1	
		C	0		4		0	
	3	A	0		1		0	
		B	1		1		0	
		C	1		2		0	

Supplementary Table 6. Virulence genes revealed by the analysis of *E. coli* O157:H7 Sakai and O157:H7 EDL933 genomes using the platform VirulenceFinder 2.0. Number of "+" indicates number of sequences of virulence factors detected in genomes.

	E. coli O157:H7 Sakai	*E. coli* O157:H7 EDL933
astA	++	++
eae	+	+
espA	+	+
espB	+	+
espF	+	+
espJ	+	+
gad	++	++
iha	+	+
iss	+++	+++
nleA	+	+
nleB	++	++
nleC	+	+
stx1A	+	+
stx1B	+	+
stx2A	+	+
stx2B	+	+
tir	+	+
stx1	+	+
stx2	+	+

Supplementary Table 7. Genomic islands, Insertion sequences and phage sequences detected in ED1 and K12 NEB DH5α genomes (*see excel file*). Genomic islands in ED1 and K12 NEB DH5α genomes detected by at least one method by IslandViewer4 (Bertelli et al., 2017) are listed respectively in excel sheets 1 and 2: the starting point, the end and the length of each genomic islands are listed in columns A, B and C. Column D indicates the gene name of Identical Protein Group of genes; columns E, F and G indicate, respectively, the locus tag of each ED1 and K12 NEB DH5α gene belonging to the genomic islands, the starting point and the ending point of genes. Functions of genes are listed in column I. Insertion sequences (IS) of ED1 and K12 NEB DH5α genomes detected by ISfinder (Siguier et al., 2006) are listed respectively in excel sheets 3 and 4: column A indicates the number of the contig in which the IS was detected; column B indicates sequences producing significant alignments and columns C, D and E indicate respectively the family, the name and the origin of the IS. Intact Phage genome sequences in ED1 and K12 NEB DH5α genomes found by PHASTER (Arndt et al., 2016) are listed in excel sheets 5 and 6: columns in the tables indicate respectively the region's length of the phage sequence, the number of total proteins of the region, the region position in ED1 and K12 NEB DH5α genomes, the phage name and its NCBI reference sequence and the GC% content.

SUPPLEMENTARY FIGURES

Supplementary Figure 1. Growth curves of ED1 (blue diamond) and DH5α (red square) strains. The slopes of the growth curves at the exponential phase were similar (slopes of the trend lines between 0.8 and 1.4 OD_{600nm} values were 0.33 and 0.30 for ED1 and DH5α strains, respectively).

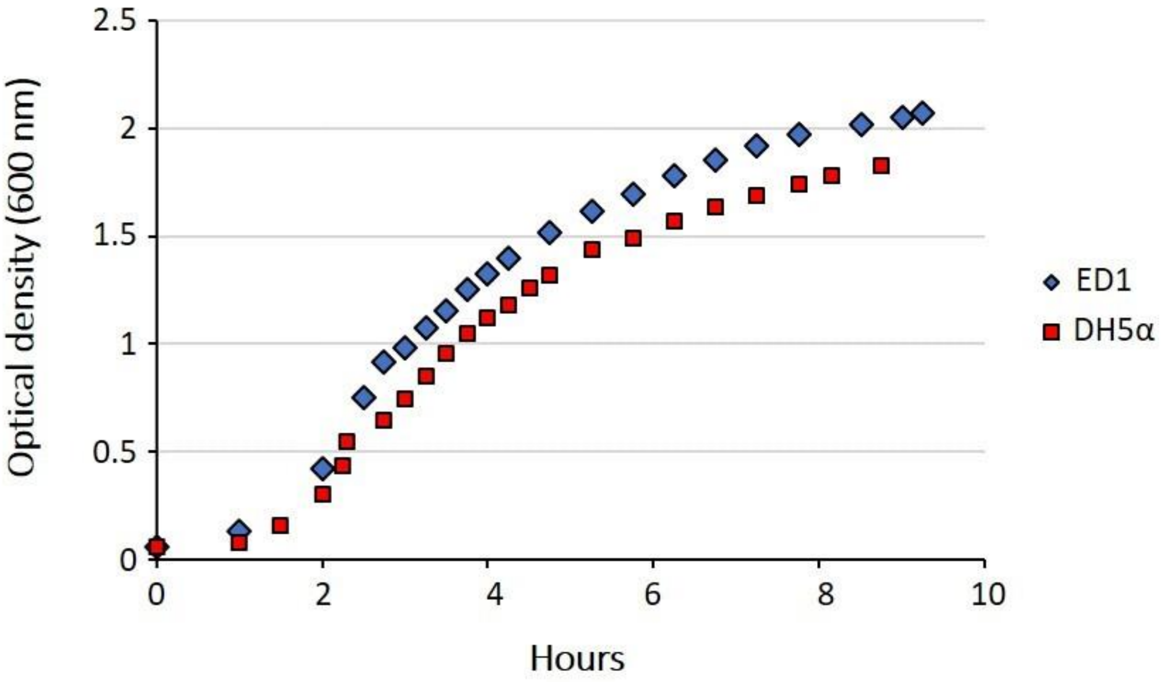

Supplementary Figure 2. Images of agarose gel electrophoresis of ITS-PCR products of (**A**) bacterial colonies (1-30) isolated from lettuce plants bacterized with *E. coli* ED1 RIF-R strain; PC = *E. coli* ED1 RIF-R positive control; (**B**) bacterial colonies (1-30) isolated from lettuce plants bacterized with *E. coli* DH5α RIF-R strain; PC = *E. coli* DH5α RIF-R positive control. NC = negative control. M = marker.

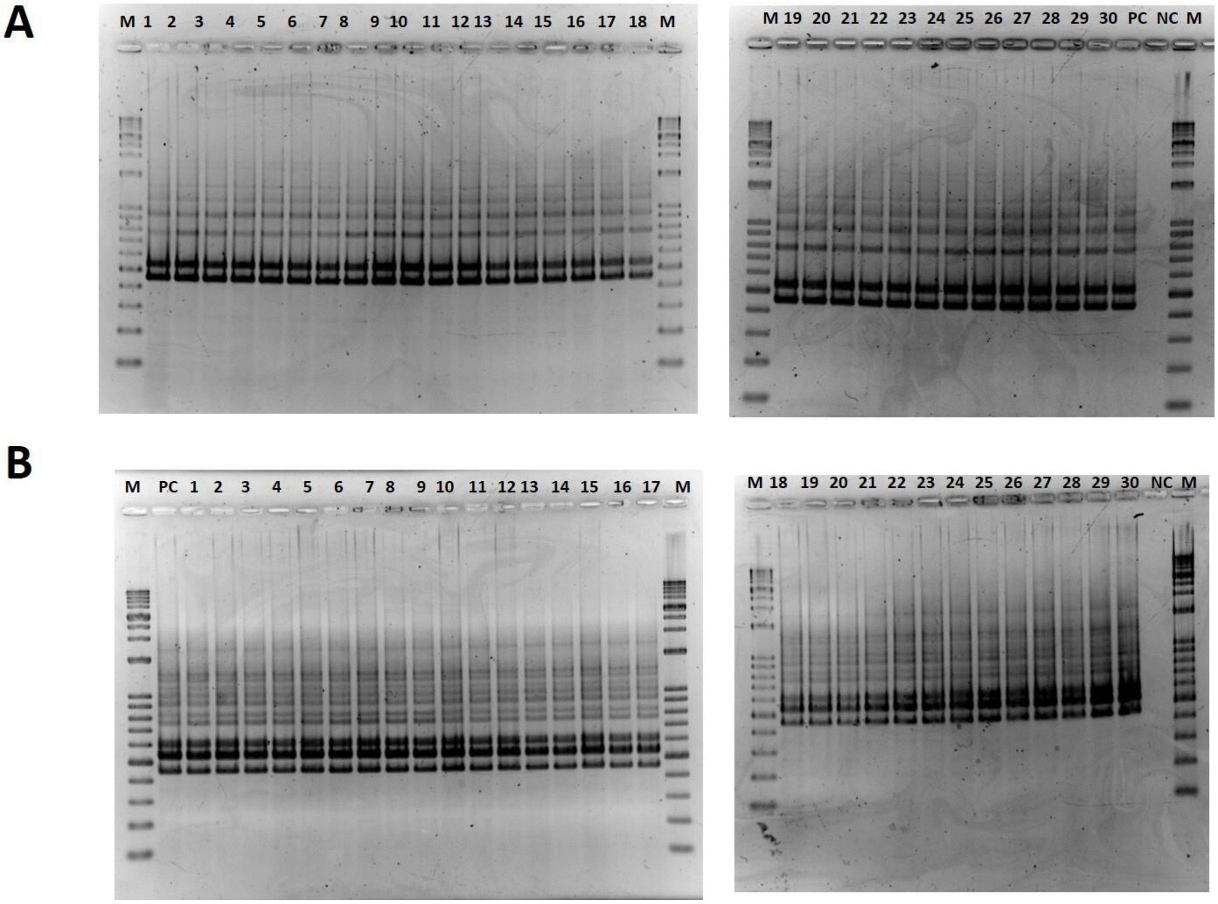

Supplementary Figure 3. Images of agarose gel electrophoresis of ITS-PCR products of bacterial colonies isolated from lettuce plants bacterized with (**A**), (**C**) *E. coli* ED1 RIF-R strain (PC = *E. coli* ED1 RIF-R positive control) and (**B**), (**D**) *E. coli* DH5α RIF-R strain (PC = *E. coli* DH5α RIF-R positive control). (**A**), (**B**) 7 days after bacterization (t1) and (**C**), (**D**) 14 days after bacterization (t2). NC = negative control. M = marker.

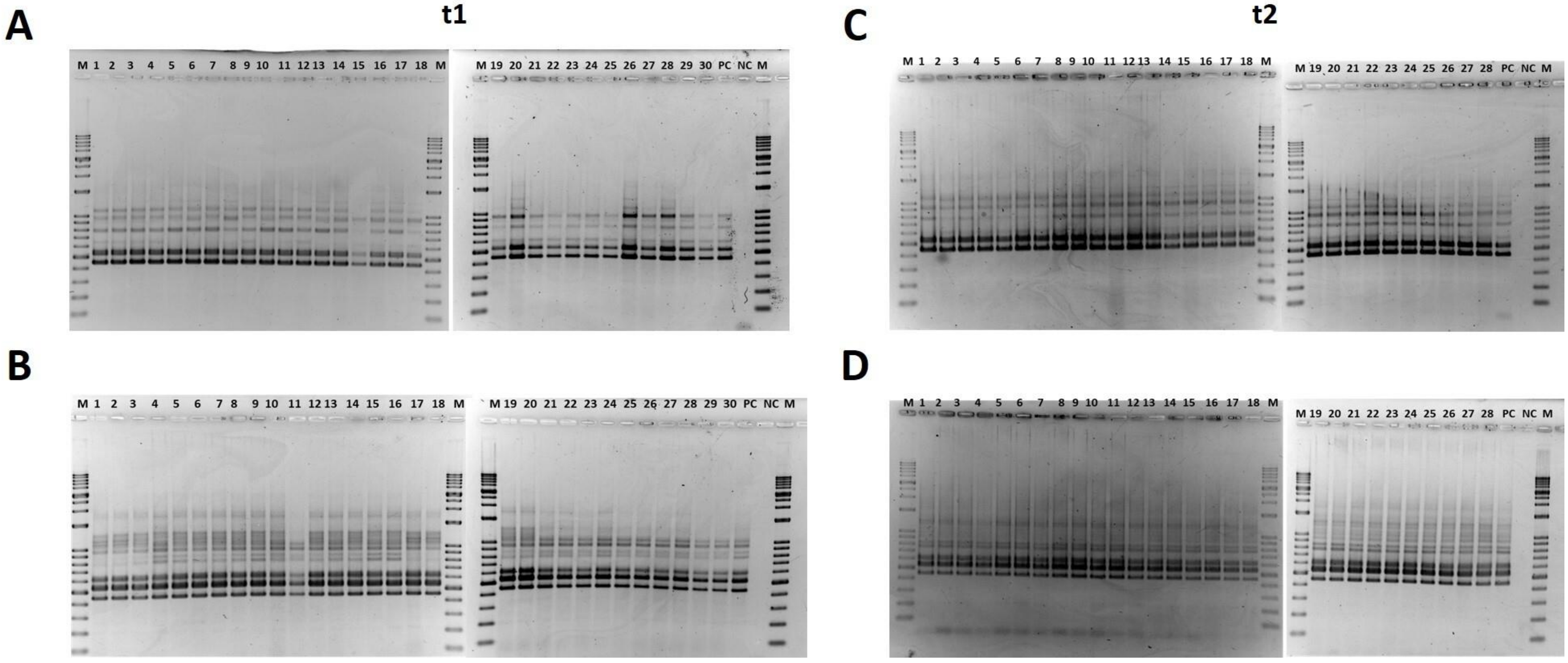

Supplement: Supplementary file 4 [file Data_Sheet_1.pdf]
